# Systematics within *Gyps *vultures: a clade at risk

**DOI:** 10.1186/1471-2148-6-65

**Published:** 2006-08-23

**Authors:** Jeff A Johnson, Heather RL Lerner, Pamela C Rasmussen, David P Mindell

**Affiliations:** 1 The Peregrine Fund, 5668 West Flying Hawk Lane, Boise, ID 83709, USA; 2 University of Michigan Museum of Zoology and Department of Ecology & Evolutionary Biology, 1109 Geddes Avenue, Ann Arbor, MI 48109, USA; 3 Michigan State University Museum and Department of Zoology, West Circle Drive, East Lansing, MI 48824-1045, USA

## Abstract

**Background:**

Populations of the Oriental White-backed Vulture (*Gyps bengalensis*) have declined by over 95% within the past decade. This decline is largely due to incidental consumption of the non-steroidal anti-inflammatory veterinary pharmaceutical diclofenac, commonly used to treat domestic livestock. The conservation status of other *Gyps *vultures in southern Asia is also of immediate concern, given the lack of knowledge regarding status of their populations and the continuing existence of taxonomic uncertainties. In this study, we assess phylogenetic relationships for all recognized species and the majority of subspecies within the genus *Gyps*. The continuing veterinary use of diclofenac is an unknown but potential risk to related species with similar feeding habits to *Gyps bengalensis*. Therefore, an accurate assessment of the phylogenetic relationships among *Gyps *vultures should aid in their conservation by clarifying taxonomic uncertainties, and enabling inference of their respective relatedness to susceptible *G. bengalensis*.

**Results:**

Phylogenetic results using mitochondrial cytB, ND2 and control region sequence data indicate a recent and rapid diversification within the genus *Gyps*. All recognized species formed monophyletic groups with high statistical support, with the exception of the Eurasian Vulture, for which specimens identified as subspecies *G. fulvus fulvescens *appear closely related to the Himalayan Vulture (*G. himalayensis*). In all analyses, the earliest divergence separated the Oriental White-backed Vulture from other *Gyps *taxa, with the next diverging taxon being either the African White-backed Vulture (*G. africanus*), or the Himalayan Vulture. All analyses supported a sister relationship between the Eurasian Vulture (*G. f. fulvus*), and Rüppell's Vulture (*G. rueppellii*), with this clade being sister to another consisting of the two taxa of "Long-billed" Vulture (*G. indicus indicus *and *G. i. tenuirostris*), and the Cape Vulture (*G. coprotheres*). These molecular phylogenies strongly support the treatment of *indicus *and *tenuirostris *as separate species, as does morphological data showing that these two taxa of similar overall size differ in proportions, especially in rostral, alar, and pedal characters. In addition, grouping of *bengalensis *and *africanus *together in the genus *Pseudogyps*, as historically proposed, is not upheld based on mitochondrial data.

**Conclusion:**

Both molecular and morphological data provide strong support for considering the "Long-billed" Vulture to be comprised of two species (*G. indicus *and *G. tenuirostris*), and further analysis is warranted to determine the taxonomic distinctiveness of *G. f. fulvescens*. Our phylogenetic analyses and conservative estimates suggest the diversification of *Gyps *taxa to be within the past 6 million years. Diclofenac susceptibility has been previously demonstrated for four *Gyps *species (*G. indicus*, *G. fulvus*, *G. africanus*, *G. bengalensis*), and the phylogenetic position of these species each forming a sister relationship with at least one of the remaining species, support concern that other *Gyps *taxa may be susceptible as well. Determining genetic and evolutionary distinctiveness for *Gyps *lineages is increasingly important as a breeding program is being established to prevent extinction.

## Background

Three Old World vulture taxa in the genus *Gyps *have recently been listed as critically endangered by The World Conservation Union [1]. These are the Oriental White-backed, or White-rumped Vulture (*G. bengalensis*) and two taxa long treated together as "Long-billed" (*G. indicus indicus *and *G. i. tenuirostris*) Vultures. All three share similar feeding behaviours, typically scavenging the soft tissues of large mammals [2,3]. This behaviour, along with their propensity to form colonies or aggregate at carcasses in large feeding groups often near human settlements, has likely contributed to their recent precipitous decline. Population declines (> 95%) of these three taxa over the past decade have been well documented. *Gyps bengalensis*, in particular, were abundant as little as ten years ago in both Pakistan and India, with nesting densities recorded as high as 12 nests/km^2 ^in Keoladeo National Park in northern India [4-9]. In fact, their decline as a significant scavenger has likely led to associated changes within their environment and has implications for human health and disease [8-11].

Oaks et al. [12] identified the apparent cause for this decline in *G. bengalensis *as diclofenac, a non-steroidal anti-inflammatory pharmaceutical commonly used to treat domestic livestock. Vultures consume diclofenac in the carcasses of treated animals, and then experience renal failure and visceral gout followed by mortality within 48 hours of ingestion [12-14]. The toxicity of this drug to non-domesticated animals other than *Gyps *vultures is not known; however, direct evidence indicates that diclofenac causes mortality in at least four of the *Gyps *taxa (e.g., *G. bengalensis*, *G. i. indicus*, *G. fulvus fulvus*, and *G. africanus*) [12,14,15].

What has been missing up to this point in *Gyps *conservation efforts is detailed consideration of their phylogeny and taxonomy. Taxonomic uncertainties remain, and resolving them can help the scientific and conservation communities in identifying and recognizing taxa at risk, in identifying their critical habitats and geographic ranges, and in promoting policies to benefit species welfare. Having well supported phylogenies and resultant taxonomies can also be useful for assessing priority in allocating limited funds for captive breeding and other conservation efforts [see [16-18]]. The extent of diclofenac susceptibility among species is not well known, though it is not unreasonable to suspect a phylogenetic component. Thus, phylogenies can help set priorities for testing susceptibility among close relatives with similar life histories.

**Figure 1:**
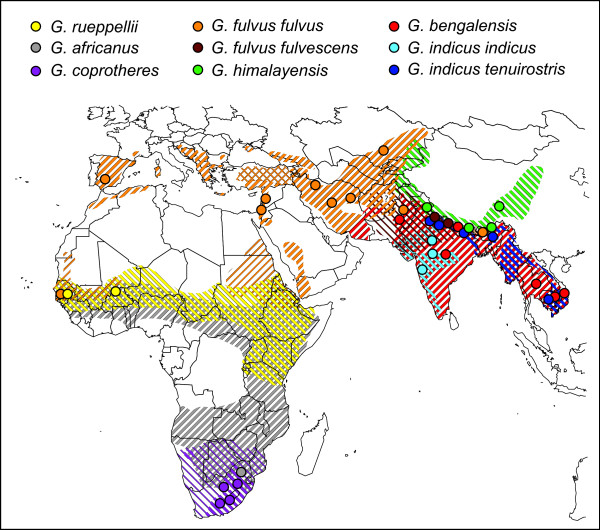
**Geographic distributions of *Gyps *and their sampled locations**. Darker diagonal lines represent year-round distributions, while thinner lines represent non-breeding distributions. Cross-hatched distributions (e.g. *G. f. fulvus *in Turkey) represent restricted breeding distributions. Uncertainty in *G. fulvus *subspecies distributions is represented by a question mark (?) at range overlap (i.e., Afghanistan). Geographic distributions determined using information provided by Mundy et al. [3], del Hoyo et al. [21], Ferguson-Lees & Christie [22], and Rasmussen & Anderton [31].

Species traditionally treated within *Gyps *are: the African White-backed Vulture (*G. africanus*), Cape Vulture (*G. coprotheres*), and Rüppell's Vulture (*G. rueppellii*) in Africa; the Oriental White-backed, or White-rumped, Vulture (*G. bengalensis*), Long-billed Vulture (*G. indicus*), and Himalayan Vulture (*G. himalayensis*) in Asia; and the Eurasian Vulture (*G. fulvus*) in Europe, Africa and Asia [19-22] (Fig. 1). As treated in these sources, *Gyps indicus, G. rueppellii*, and *G. fulvus *are polytypic. In a number of earlier references, *G. bengalensis *and *G. africanus *have been classified together as a separate genus, *Pseudogyps*, due primarily to a smaller body size and a reduced number of rectrices (12 vs. 14) compared to other *Gyps *taxa [3,23-25]. In addition, proposals have been made to consider the "Long-billed" Vulture as two separate species based on morphological differences [26-31], and the taxonomic status of the two subspecies of Eurasian Vulture (*G. f. fulvus *and *G. f. fulvescens*), as well as their characteristics and geographic distribution are unclear. To date, taxonomic relationships among *Gyps *taxa, including subspecies relationships, remain to be assessed with molecular sequence characters, and the validity of *fulvescens*, which has not been considered in recent times [27,28,32], clearly warrants further study.

Here we assess phylogenetic relationships among all currently recognized *Gyps *species using molecular methods. Some taxa from this genus have been incorporated previously in molecular phylogenetic studies [33-35]; however, none of these studies included all recognized *Gyps *taxa. Indeed, *G. indicus *(including *tenuirostris*), *G. himalayensis*, and *G. f. fulvescens *have never been included in a molecular phylogenetic study. Therefore, this is the first attempt to ascertain *Gyps *systematics based on samples of all recognized species using molecular techniques. We include morphological data and analyses to further investigate taxonomic status of the two "Long-billed" Vulture taxa (*G. i. indicus *and *G. i. tenuirostris*).

## Results

### Sequence characteristics

Among 60 representative individuals from the genus *Gyps *using complete mitochondrial (mt) cytochrome B (cytB) sequence data (1024 bp), 27 unique haplotypes were distinguished based on 81 variable sites (76 transitions and five transversions). Combined analysis of 2092 characters from mt cytB and NADH dehydrogenase subunit 2 (ND2), from a smaller set of individuals (n = 20), identified 16 unique haplotypes based on 131 variable sites (121 transitions and 10 transversions) among ingroup taxa. For 400 bp of mt control region (CR), 15 unique haplotypes were identified for 20 individual *Gyps *vultures, including 29 variable sites (25 transitions, four transversions and one indel). When CR was combined with corresponding cytB and ND2 sequence data, 19 unique haplotypes based on 160 variable sites were observed among 20 individual *Gyps *vultures. Uncorrected percent sequence divergence between taxa was similar across loci with CR showing slightly higher divergence estimates; however, these differences were taxon specific with cytB or ND2 showing higher divergence estimates in some cases (Table 1).

**Table 1:** Observed percent uncorrected (*p*) pairwise sequence divergences. Minimum and maximum observed percent uncorrected (*p*) pairwise sequence divergences for each locus including the combined dataset (below and including the diagonal) and number of nucleotide differences among pairwise comparisons for the combined dataset (above the diagonal).

	*indicus*	*tenuirostris*	*coprotheres*	*rueppellii*	*fulvus*	*fulvescens*	*himalayensis*	*africanus*	*bengalensis*	outgroup
*G. i. indicus*										
cytB	0.0									
ND2	0.0									
CR	0.3									
CR+ND2+cytB	0.0	25–27	24–26	27–30	29–32	56–57	54–55	60–64	47–51	228–249
*G. i. tenuirostris*										
cytB	0.8–0.9	0.0–0.1								
ND2	1.1–1.3	0.2								
CR	1.3–1.5	0.0								
CR+ND2+cytB	1.0–1.1	0.1	26–30	30–34	34–38	59–61	57–59	64–66	54–58	233–251
*G. coprotheres*										
cytB	0.9–1.1	1.5–1.8	0.0–0.6							
ND2	0.7–0.8	0.6–0.8	0.0							
CR	1.8–2.0	1.3	0.0							
CR+ND2+cytB	1.0–1.1	1.0–1.2	0.2	30–32	29–35	58–59	56–57	60–68	55–57	226–244
*G. rueppellii*										
cytB	0.5–0.6	1.1–1.3	1.2–1.5	0.1						
ND2	1.1–1.2	0.9–1.2	0.6–0.7	0.1						
CR	2.5–2.8	2.3	2.5–3.0	0.5						
CR+ND2+cytB	1.1–1.2	1.2–1.4	1.2–1.3	0.2	22–24	46–48	44–46	58–60	51–56	228–252
*G. f. fulvus*										
cytB	0.5–0.9	0.9–1.5	1.1–1.8	0.6–1.1	0.0–0.6					
ND2	1.2–1.5	1.0–1.4	0.7–0.9	0.7–1.0	0.2–0.5					
CR	2.5–3.0	2.8–3.0	2.8–3.0	1.5–2.3	0.0–0.3					
CR+ND2+cytB	1.2–1.3	1.4–1.5	1.2–1.4	0.9–1.0	0.1–0.2	51–53	49–51	61–65	53–57	239–254
*G. f. fulvescens*										
cytB	1.8–2.0	2.4–2.6	2.4–2.7	1.9–2.1	1.8–2.5	0.6				
ND2	2.3–2.4	2.2–2.3	1.8	1.8–1.9	2.1–2.4	0.0				
CR	2.5–2.8	2.3	3.0	1.5	1.5–1.8	0.0				
CR+ND2+cytB	2.2–2.3	2.4–2.5	2.4	1.9	2.1	0.0	1–2	68–71	59–62	250–262
*G. himalayensis*										
cytB	1.9–2.0	2.5–2.7	2.5–2.7	2.0–2.1	1.9–2.5	0.1–0.6	0.0–0.2			
ND2	2.2–2.3	2.1–2.3	1.7	1.7–1.8	2.0–2.3	0.1	0.0			
CR	2.5–2.8	2.3	3.0	1.5	1.5–1.8	0.0	0.0			
CR+ND2+cytB	2.2	2.3–2.4	2.3	1.8–1.9	2.0–2.1	0.0–0.1	0.0	66–69	57–60	248–262
*G. africanus*										
cytB	1.4–1.7	2.0–2.1	2.1–2.6	1.8–2.1	1.7–2.4	2.3–2.9	2.6–2.9	0.1–0.2		
ND2	3.0–3.1	2.8–3.1	2.4–2.5	2.4–2.6	2.7–3.1	2.5–2.6	2.4–2.5	0.1		
CR	3.0–3.5	3.0	2.8–3.8	2.8–3.3	3.0–3.3	3.3	3.3	1.5		
CR+ND2+cytB	2.4–2.6	2.6–2.7	2.4–2.8	2.4	2.5–2.6	2.8–2.9	2.7–2.8	0.3	59–65	236–252
*G. bengalensis*										
cytB	1.7–1.9	2.3–2.6	2.4–2.7	1.9–2.2	1.8–2.6	2.0–2.8	2.4–2.8	2.2–2.6	0.0–0.5	
ND2	2.3–2.4	2.1–2.3	1.8	1.8–1.9	1.9–2.1	2.1	2.0	2.2–2.3	0.0	
CR	1.3–2.0	2.0–2.3	2.8–3.3	3.3–3.8	3.3–3.5	2.8–3.3	2.8–3.3	3.3–4.0	0.0–0.8	
CR+ND2+cytB	1.9–2.1	2.2–2.3	2.2–2.4	2.1–2.3	2.1–2.3	2.4–2.5	2.3–2.4	2.4–2.6	0.0–0.2	224–243
outgroup taxa^1^										
cytB	7.5–9.1	7.7–9.6	7.8–9.5	7.2–9.6	7.4–9.8	7.4–9.7	7.6–9.8	7.7–9.6	7.1–9.2	4.0–9.8
ND2	9.2–10.2	8.7–10.2	8.6–9.8	8.6–9.9	8.8–10.4	8.6–10.6	8.5–10.5	8.3–10.1	8.0–9.4	5.2–10.6
CR	11.3–14.1	11.8–14.3	11.3–13.8	12.8–15.3	13.5–15.3	13.0–15.6	13.0–15.6	12.8–16.1	11.8–15.6	10.8
CR+ND2+cytB	9.2–10.0	9.4–10.1	9.2–9.8	9.3–10.1	9.7–10.3	10.1–10.5	10.0–10.6	9.6–10.1	9.0–9.8	10.1

^1 ^outgroup taxa for analyses including control region (CR) are restricted to two taxa instead of five (see methods)

Nucleotide composition varied slightly between cytB and ND2 with both loci displaying lower levels of guanine (13 and 10%, respectively) and higher levels of cytosine (34 and 37%) nucleotides than expected by chance. CR also possessed lower levels of guanine (19%); however, it differed from cytB and ND2 in showing higher levels of thymine (32%) nucleotides. Tests for departure from homogeneity in base frequencies across taxa with and without uninformative mt characters were not significant for all three loci analyzed separately or combined (χ^2^, *P *> 0.05).

### Phylogenetic analyses

**Figure 2:**
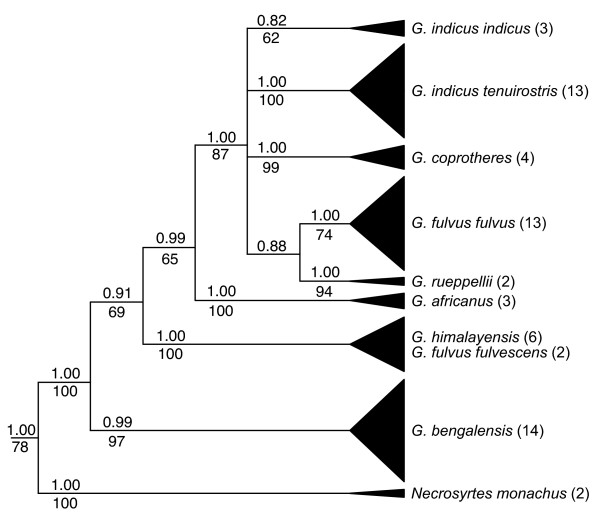
**Phylogeny for *Gyps *taxa based on mt cytB**. The topology shown is the Bayesian inference majority rule tree. MP bootstrap nodal support values (greater than 50%) are below branches and the Bayesian posterior probability values are above. Numbers of individuals sampled per taxon are indicated in parentheses. Three additional outgroup species used in the analysis (*S. calvus*, *T. tracheliotos*, and *T. occipitalis*) are not shown.

**Figure 3:**
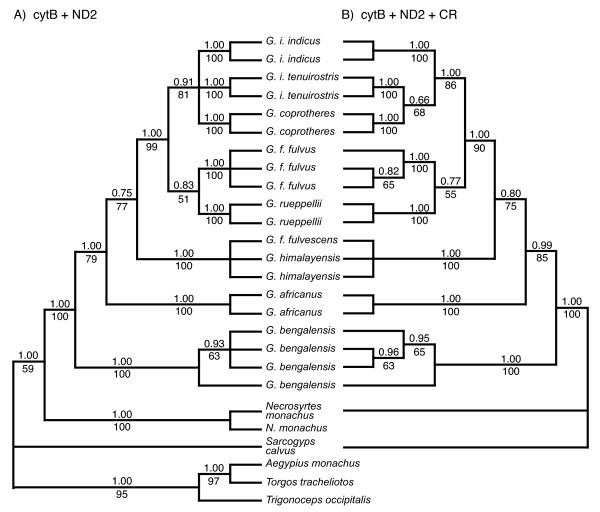
**Phylogeny for *Gyps *taxa based on combined mt ND2 and cytB datasets (A) and combined CR, ND2, and cytB datasets (B)**. The topologies shown are the Bayesian inference majority rule trees, and these are congruent with MP analyses as well. MP bootstrap nodal support values (greater than 50%) are below the branches and Bayesian posterior probabilities are above.

The AIC identified the GTR+G model of sequence evolution [36] for analyses of both cytB and ND2. When partitioned by codon position, GTR+G, HKY+I, and HKY models were selected for each successive codon position (1^st^, 2^nd^, and 3^rd^, respectively) for cytB, and HKY+G, HKY+I, and GTR+I models were selected for each successive codon position for ND2. The CR was analyzed with equal weights among characters in all analyses. The same topology was found in both MP and Bayesian analyses irrespective of utilizing codon positions for the Bayesian cytB analyses and also for each of the two multi-locus datasets; however, the mixed models provided increased support indices at most nodes for all data sets, and therefore, only the support indices while utilizing codon partitions are shown for the Bayesian results (Figs. 2, 3).

Regardless of dataset (single or multi-locus), monophyly of the genus *Gyps *and each species was strongly supported with high bootstrap support and posterior probabilities for each clade (Figs. 2, 3). The high number of nucleotide differences consistently observed between taxa further highlight these diagnostic relationships (Table 1). No geographic partitioning was observed within species or subspecies possessing large samples sizes (i.e., *G. bengalensis *and *G. f. fulvus*; data not shown). However, within *G. indicus*, the Long-billed (*G. i. indicus*) and the Slender-billed (*G. i. tenuirostris*) vultures formed two separate monophyletic clades with high statistical support. Similarly, representative individuals of the two subspecies of Eurasian Vulture, *G. f. fulvus *and *G. f. fulvescens *were phylogenetically distinct; however, they were not placed as sister taxa. Both *fulvescens *samples clustered with the Himalayan Vulture (*G. himalayensis*; Figs. 2, 3). One of the two birds identified as *G. f. fulvescens *had an identical CR haplotype and differed by a single nucleotide from four of the six and all of the *himalayensis *haplotypes in cytB and ND2, respectively (Table 1; Additional file 1). DNA extractions for these taxa were conducted separately with multiple independent PCR amplifications to verify these results and to help rule out the possibility of contamination.

There were a few differences in sister relationships among *Gyps *species when comparing results from different datasets (i.e., whether analyses were conducted for each locus separately or combined with others; Figs. 2, 3). The CR analysis identified monophyletic species similar to cytB and ND2; however, further resolution was limited with all species forming a single polytomy (tree not shown). When ND2 was analyzed separately (tree not shown), its topology was identical to that provided by the combined cytB and ND2 results (Fig. 3), while the topology for cytB alone differed from results given by the multi-locus datasets. In all analyses, the earliest divergence separated *G. bengalensis *from all other *Gyps *taxa; however, whether the next divergence is for *G. africanus *or *G. himalayensis*/*G. f. fulvescens *varies by dataset analyzed, with *G. africanus *divergence supported as the second divergence within *Gyps *by cytB and ND2 combined as well as the cytB, ND2 and CR combined dataset. All analyses supported a sister relationship between *G. f. fulvus *and *G. rueppellii*, with this clade sister to a clade consisting of *G. i. indicus*, *G. i. tenuirostris*, and *G. coprotheres*, and with the latter taxa forming a polytomy in the combined cytB and ND2 analyses without CR (Fig. 3A). In the multi-locus dataset including the CR (Fig. 3B), *G. i. tenuirostris *and *G. coprotheres *are posited as sisters with only weak statistical support.

### Long-billed Vulture morphological analyses

Although the two taxa long classified as subspecies of "Long-billed" Vulture (*G. i. indicus *and *G. i. tenuirostris*) are similar in overall size, they differ markedly in proportions (Table 2). The rostrum of *tenuirostris *is much longer than that of *indicus *(as shown by culmen length and bill length from gape), while in *indicus *the rostrum is deeper and broader (as shown by bill width, bill depth, and maxilla depth). The longer skull and mandibular symphysis of *tenuirostris *is probably also a reflection of its relatively longer bill. The nostrils (nares length) of *indicus *are much longer than *tenuirostris *(reflecting the ovate shape of the nostril of *indicus *vs. the round nares of *tenuirostris*). In wing proportions, the "arm" (ulna length) and alula of *tenuirostris *are longer than for *indicus *while the "hand" (wing length) is longer in *indicus*. Lengths of individual primaries measured from the carpal joint did not differ significantly between the taxa and are not presented here. For the pes, most elements of *tenuirostris *are significantly longer than those of *indicus*, with the exception of the claws of digits 1 and 2, whereas pedal elements of *indicus *are proportionately more similar to those of *tenuirostris *in width and breadth measures.

**Table 2:** External measurements (mm) of *Gyps indicus *and *G. tenuirostris *presented as mean ± SD (n)

Variable	*indicus*	*tenuirostris*
Skull length***	129.18 ± 3.64 (14)	136.04 ± 4.36 (13)
Culmen l**	67.57 ± 2.84 (17)	69.76 ± 1.66 (17)
Bill w***	20.88 ± 0.84 (19)	19.82 ± 0.72 (21)
Bill d*	30.93 ± 1.49 (13)	29.64 ± 1.27 (16)
Maxilla d**	24.46 ± 1.37 (19)	23.34 ± 0.95 (19)
Nares l***	13.01 ± 1.64 (20)	9.89 ± 1.02 (21)
Gape w	34.17 ± 1.60 (20)	34.83 ± 2.18 (19)
Bill l from gape***	70.80 ± 3.72 (18)	75.47 ± 2.17 (18)
Mandibular symphysis l***	26.53 ± 1.55 (20)	29.39 ± 1.48 (20)
Tail l	240.75 ± 9.05 (20)	241.45 ± 10.19 (20)
Outer rectrix l*	231.47 ± 9.93 (19)	224.22 ± 8.98 (18)
Ulna l**	313.27 ± 10.58 (15)	326.33 ± 14.98 (18)
Alula l***	214.85 ± 7.01 (20)	227.90 ± 5.35 (20)
Wing l (flattened)	642.40 ± 15.73 (15)	637.73 ± 13.32 (15)
Tarsus l***	107.13 ± 4.11 (20)	114.88 ± 5.862 (19)
Tarsus proximal b*	25.19 ± 1.58 (20)	26.40 ± 1.74 (19)
Tarsus minimum b	14.10 ± 0.92 (17)	14.51 ± 1.00 (17)
Tarsus distal b**	26.38 ± 1.48 (19)	28.04 ± 1.76 (20)
Pes digit 1 l***	34.00 ± 1.26 (19)	37.75 ± 2.79 (20)
Pes digit 1 claw l	30.26 ± 1.58 (18)	30.05 ± 1.39 (20)
Pes digit 2 l***	47.01 ± 2.42 (19)	50.66 ± 3.47 (20)
Pes digit 2 claw l	30.40 ± 1.65 (19)	31.24 ± 1.68 (20)
Pes digit 3 l***	93.54 ± 3.84 (16)	103.22 ± 3.90 (19)
Pes digit 3 claw l***	27.99 ± 2.04 (16)	31.15 ± 1.84 (20)
Pes digit 3 claw w***	6.75 ± 0.38 (18)	6.32 ± 0.36 (20)
Pes digit 3 claw d***	7.26 ± 0.34 (18)	7.78 ± 0.40 (20)
Pes digit 4 l***	55.32 ± 2.58 (18)	61.38 ± 4.05 (18)
Pes digit 4 claw l*	23.95 ± 1.52 (18)	24.93 ± 1.30 (19)

Sexes pooled. Significance levels: *, P ≤ 0.05; **, P ≤ 0.01; ***, P ≤ 0.001. L, length; w, width; d, depth; b, breadth.

**Figure 4:**
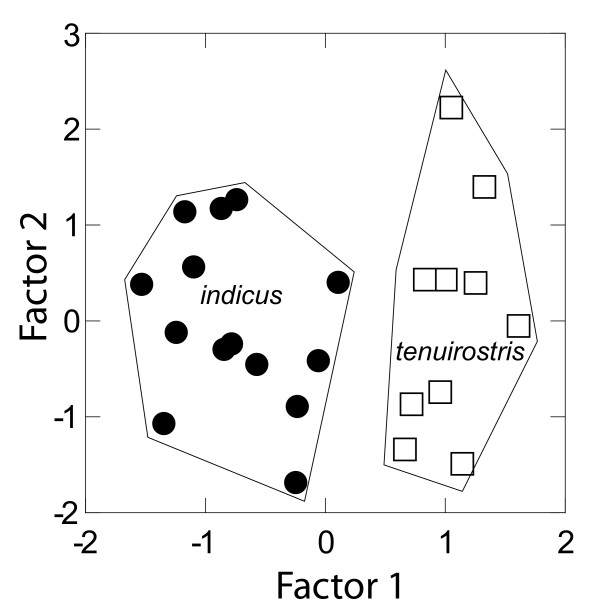
**Plot for scores of Principal Components Analysis Factors 1 and 2 for external mensural characters of *Gyps indicus *and *G. tenuirostris***. *Gyps indicus *and *G. tenuirostris *are significantly different (*P *≤ 0.001) on Factor 1. Individuals with strongly positive scores on Factor 1 are *tenuirostris*, which have longer tarsi and toes, but narrower and longer rostra relative to *indicus*.

In a Principal Components Analysis (PCA), Factor 1 was a highly significant (*P *≤ 0.001) shape axis distinguishing *indicus *and *tenuirostris *specimens (Table 3). Variables with high positive loadings on PCA Factor 1 were lengths of culmen, bill from gape, mandibular symphysis, alula, tarsus, tarsus proximal, tarsus distal, toes (pes digits), and depth of the claw of digit III. These variables contrasted with the strongly negatively loading nares length, and to a lesser extent with bill width, outer rectrix length, and width of the claw of digit III. Although the first six factors had eigenvalues above 1, component loadings of *indicus *and *tenuirostris *were significantly different only on Factor 1. Nevertheless, on this axis they were significantly different and readily distinguished (Fig. 4).

**Table 3:** Summary results for principal components analysis of external measurements of *Gyps indicus *and *G. tenuirostris*.

Factor component loadings						
Variable	1	2	3	4	5	6
Culmen l	0.75	-0.21	0.22	0.45	0.00	-0.20
Bill w	-0.38	0.56	0.41	0.35	0.20	-0.17
Maxilla d	-0.17	0.49	0.28	0.48	0.28	0.23
Nares l	-0.80	0.29	0.06	0.04	0.34	-0.01
Gape w	-0.01	0.30	0.07	0.66	-0.01	0.12
Bill l from gape	0.79	0.15	-0.08	0.34	-0.20	0.01
Mandibular symphysis l	0.74	0.33	0.32	0.11	0.13	0.10
Tail l	0.01	-0.07	-0.69	-0.47	0.18	0.25
Outer rectrix l	-0.29	-0.29	0.80	0.12	-0.18	0.20
Alula l	0.72	0.19	0.06	-0.17	-0.04	0.35
Tarsus l	0.72	0.25	-0.12	-0.24	0.11	0.22
Tarsus proximal b	0.60	-0.02	0.57	-0.03	0.12	-0.40
Tarsus minimum b	0.33	-0.12	0.42	-0.41	0.01	0.28
Tarsus distal b	0.63	-0.30	0.09	0.15	-0.30	-0.45
Pes digit I l	0.64	0.42	-0.11	-0.17	0.28	0.02
Pes digit I claw l	-0.04	-0.71	0.01	-0.09	0.61	0.09
Pes digit II l	0.56	0.41	-0.11	-0.38	0.37	-0.36
Pes digit II claw l	0.15	-0.47	-0.24	0.51	0.21	0.51
Pes digit III claw w	-0.36	-0.15	0.02	0.18	0.62	-0.32
Pes digit III claw d	0.71	-0.46	0.20	0.22	-0.07	-0.08
Pes digit IV l	0.75	0.28	-0.26	0.09	0.11	0.22
Pes digit IV claw l	0.53	-0.43	-0.21	0.09	0.49	-0.03
Summary statistics						
Eigenvalues	6.78	2.76	2.30	2.17	1.76	1.43
Percent variance explained	30.81	12.53	10.46	9.87	8.00	6.48
*P*	***	ns	ns	ns	ns	ns

Significance levels (ns, P > 0.05; ***, P ≤ 0.001) from two-sample t-test between component loadings for *indicus *and *tenuirostris*. Abbreviations for variables are as in Table 3.

## Discussion

Our objective in this study is to resolve phylogeny and taxonomic uncertainties for *Gyps *taxa, in order to inform current conservation efforts. By using museum specimens as DNA sources along with tissues obtained from the field, we sampled representatives of all generally recognized *Gyps *taxa with emphasis on those geographically distributed in south Asia; the primary area experiencing recent, drastic population declines. Our analyses support two changes to the traditional taxonomy for *Gyps*. First, two individuals identified as *G. f. fulvescens *were most closely related to *G. himalayensis *(Figs. 2, 3). Relatively high divergence estimates among all *G. fulvus *individuals (1.5–2.5%, Table 1) and relatively low divergence estimates between *G. f. fulvescens *and *G. himalayensis *(0.0–0.6%) reflect this phylogenetic result. Additional sampling and analyses for *G. f. fulvescens *are needed to verify these results. Second, our analyses based on both morphological and molecular data indicate the phylogenetic distinctiveness of the Long-billed and the Slender-billed Vultures, supporting their taxonomic treatment as distinct species (e.g., *G. indicus *and *G. tenuirostris*, respectively) as recommended previously [29-31]. Mensural analyses show that *indicus *and *tenuirostris *differ significantly in proportions, especially of the head, wing, and pes (Table 2), and all individuals in each taxon are clearly separated on at least one axis in a PCA (Table 3). In our molecular analyses, pairwise sequence divergences between *G. indicus *and *G. tenuirostris *are similar to their respective divergence estimates from *G. coprotheres *(Table 1), and to those reported between various other broadly recognized species within the family Accipitridae [35]. These results highlight the utility of molecular phylogenetic methods in identifying independent evolutionary lineages within a group that has a long history of taxonomic uncertainty [3,27,28,32-35,37-39], and, furthermore, help identify and resolve problematic specimen identifications (i.e., *fulvescens*; see also [17,40]).

The phylogenetic relationships found among *Gyps *vultures were largely the same for the different methods and mt datasets. Despite our finding of monophyly for the majority of *Gyps *species, relatively small sequence difference estimates (0.5–3.8%; Table 1) separating some named species made determination of sister relationships difficult, and multiple relationships were unresolved due to low nodal support. This suggests that the *Gyps *study taxa stem from relatively rapid and recent diversification events. If we use a generally supported avian mtDNA divergence rate for coding regions ranging from 1.6 to 5.0% change per million years (see [41]), our mt cytB and ND2 sequence divergence estimates (GTR+G; 0.8–3.4%), indicate that the radiation of *Gyps *vulture study species occurred 0.2 to 2.1 million years ago. These estimates must be considered with caution as they assume clock-like rates of sequence change, which is known to be violated in comparisons of some avian taxa and genes (e.g. [42-45]). However, we were not able to reject a hypothesis of clock-like behavior for our particular *Gyps *sequence dataset using a log likelihood ratio test (-ln L_clock _= 3743.13, -ln L_non-clock _= 3731.94; 2Δln L = 22.38; d.f. = 18; P > 0.05). Even if we assume that the above divergence rates are too high (see [45]), a lower rate (e.g., 0.6% per million years) still yields divergence times that are quite recent (< 5.7 million years).

These divergence estimates do not necessarily correspond with geographic proximity or the current distributions of species. For example, divergence estimates between *G. indicus *and both *G. coprotheres *and *G. rueppellii *are relatively low (0.9–1.3%; cytB & ND2 combined), yet the species compared occupy different continents. In contrast, divergence estimates between species with geographically proximate distributions, *G. coprotheres *and *G. africanus *in Africa and *G. i. tenuirostris *and *G. himalayensis *in South Asia (see Fig. 1) are relatively high (2.9–3.2% and 2.8–3.1%, respectively).

The historic radiation of this genus likely evolved in environmental conditions that no longer exist to the same extent throughout their current distributions. *Gyps *species are unique among Old World vultures in that they feed exclusively as scavengers, whereas other vultures are also known to kill their prey on occasion or, rarely, to feed on fruits (i.e., *Gypohierax angolensis*; [2,3,21]. This specialization in feeding behavior among *Gyps *vultures is thought to have evolved due to their close association with ungulate populations, particularly migratory populations in Africa and Asia. In fact, the observed temporal and geographic diversification of *Gyps *vultures coincides with the diversification of Old World ungulates, especially in the family Bovidae [46-50], and the expansion of grass-dominated ecosystems in Africa and Asia (see [51]). These close associations likely played a significant role in the adaptation and rapid diversification of *Gyps *vultures. Indeed, Houston [2] proposed that their large body size and ability to soar over large distances in search for food are related to the associated migrant distributions and seasonal fluctuations in mortality of ungulates, and that they have consequently become incapable of actually killing their own prey (see also [52]).

## Conclusion

Both molecular and morphological data provide strong support for considering the "Long-billed" Vulture (*G. indicus*) to be comprised of two species, the Long-billed Vulture (*G. indicus*) and the Slender-billed Vulture (*G. tenuirostris*), with both considered critically endangered by the IUCN [1]. We found non-monophyly for our set of Eurasian Vultures, with both *G. f. fulvescens *individuals appearing more closely related to *G. himalayensis *than to *G. f. fulvus*, suggesting a topic for further analysis. Our phylogenetic analyses indicate the oldest divergence among *Gyps *species to be between *G. bengalensis *and the others, and conservative estimates suggest the diversification of *Gyps *taxa to be within the past 6 million years.

The scavenging lifestyle of *Gyps *vultures and the decline of their historical food sources has likely contributed to their increased dependence on habitats heavily impacted by humans (see [3]). Many *Gyps *vulture populations have become increasingly dependent on domesticated animals, especially cattle, and this has contributed to their catastrophic decline in Pakistan and India, due to their secondary exposure to the veterinary pharmaceutical drug diclofenac (see [12,13,15,53]). *Gyps bengalensis *was fairly recently described as the most abundant large bird of prey in the world [4], yet, in as little as ten years, this species has become exceedingly difficult to find in the wild (see [54] for current trends).

Determining genetic and evolutionary distinctiveness for *Gyps *lineages is increasingly important as a captive-breeding program is being established to prevent *G. bengalensis *extinction and other *Gyps *taxa are considered to be at risk or of uncertain status. Diclofenac susceptibility has been previously demonstrated for four *Gyps *species (*G. indicus, G. fulvus, G. africanus, G. bengalensis *[12-15]), and the relative recency of diversification and the phylogenetic position of these four known susceptible species each forming a sister relationship with at least one of the remaining taxa in this genus, support concern that the other *Gyps *taxa may be susceptible as well (see also [11,14]). The most obvious long-term solution to prevent their extinction is the immediate removal of diclofenac as a veterinary drug for domestic livestock. A recent study reported on findings suggesting that an alternative drug called meloxicam may serve as a surrogate to diclofenac without causing harm to *Gyps *vultures [11]. Fortunately, India has since banned the manufacture and use of diclofenac [55]; however, the drug is still available for veterinary use in Pakistan and vulture populations continue to decline.

## Methods

### Taxon sampling, DNA extraction, amplification and sequencing

To infer phylogenetic relationships among *Gyps *taxa, a total of 60 individuals were sampled throughout a large proportion of their geographic range with emphasis on south Asia (Fig. 1; additional file 1). At least two individuals were sampled from each of the recognized species or subspecies [19-21] with some taxa having as many as 14 representative individuals depending on the particular locus utilized in the analyses (see additional file 1). In an attempt to prevent confusion, we have elected to use "vultures" for the common names used herein, rather than those often used in which certain species are referred to as "griffons". This is because our phylogenetic results clearly demonstrate that "griffons" *sensu lato *[[20,21]; but see [3,22]] are not a monophyletic group unless, of course, it is restricted to a single taxon. Outgroup taxa for mt cytB and ND2 phylogenetic analyses included the Hooded Vulture (*Necrosyrtes monachus*), Red-headed Vulture (*Sarcogyps calvus*), Monk Vulture (*Aegypius monachus*), Lappet-faced Vulture (*Torgos tracheliotos*), and White-headed Vulture (*Trigonoceps occipitalis*) (see [35]). For analyses including mt CR sequence data, the outgroup taxa were restricted to *N. monachus *and *S. calvus *due to difficulties in alignments and presence of indels associated with the other outgroup taxa.

Total genomic DNA was extracted from blood or from toe-pad tissue for museum specimens using a DNeasy Tissue Extraction Kit (QIAGEN Inc.). All work with museum samples was conducted in a facility used only for ancient DNA work at the University of Michigan Museum of Zoology, with protocols developed for ancient DNAs (e.g., [56,57]). PCR amplifications were performed with Platinum Taq (Invitrogen) using primers designed for mt cytB, ND2 (L5219/H5766 and L5758/H6313; [58]), and approximately 400 basepairs (bp) from the 5' end of the control region (Table 4). We obtained nucleotide sequences for cytB, ND2 and control region from 67, 26, and 22 representative individuals, respectively, including outgroup taxa (see additional file 1). Potential contamination was carefully monitored through the use of multiple extraction and PCR controls. PCR products were directly sequenced in both directions with ABI Big Dye Terminator chemistry, resolved on an ABI 3730 automated sequencer (Applied Biosystems), and deposited in GenBank [GenBank:DQ908960–DQ909007].

**Table 4:** Primers used for the amplification of mt cytB and control region in *Gyps *taxa

Primer ID	Sequence (5-3')
Control region	
GbCR1.L	TGT ACA TTA CAC TAT TTG CCC CAT A
GbCR2.H	GCA GGG GGA AAG TAA GAT CC
cytB	
L14996.gyps^1^	ATC TCH GCH TGA TGA AAY TTY GG
H379.gyps	AGG GTT TGT CCG ATG TAT GG
L312.gyps	CGT CCT ACC ATG AGG ACA AA
H15646.gyps^1^	GGG GTG AAG TTT TCT GGG TC
L15556.gyps^1^	CTG YGA CAA AAT CCC ATT CCA
H821.gyps	GCG YTG TTT GGA YTT GTG TA
L749.gyps	GCR TAC GCT ATT CTA CGC TCA
H16064.gyps^1^	CTT CAS TYT TTG GTT TAC AAG ACC

^1 ^modified from sequences given in Sorenson *et al*. [58].

### Alignment and phylogenetic analysis

Sequences were aligned by eye. No indels were observed in cytB or ND2, and the few indels observed in the control region were readily resolved in alignments, excluding three of the outgroup taxa (see above). We used both maximum parsimony (MP) and Bayesian inference using Markov chain Monte Carlo (MCMC) sampling approaches to reconstruct phylogenies. Analyses were conducted using samples for each locus separately and combined (cytB/ND2 and cytB/ND2/CR). MP trees were inferred using PAUP* 4.0b10 [59], and all character-state changes were equally weighted. All MP analyses were heuristic with starting trees obtained by random taxa addition with 100 replicates, TBR branch swapping, and support values for clades were calculated from 1000 bootstrap replicates.

Bayesian analyses were implemented using MrBayes v. 3.0B4 [60,61]. A number of recent studies have shown that partitioning data by codon position or gene region can produce less biased posterior probability estimates and allow a better fit between particular models and their corresponding sequence data [62-64]. Therefore, the best-fit model of evolution was determined by Akaike information criterion (AIC) in Mr. ModelTest v. 2.0 [36] with best-fit models assessed using information from codon nucleotide positions for cytB and ND2 separately, and equal weights among nucleotide positions given for the CR (see Results). All Bayesian analyses were run for six million generations, of which the first 50 000 generations were discarded before determining posterior branch probabilities. Four chains in the Bayesian MCMC analyses were used in each of four independent runs. Each of the independent runs converged on similar optimal log likelihood scores and identical tree topologies. The criterion of monophyly was used for diagnosing distinctive evolutionary units and for assessing taxonomic associations.

### Long-billed Vulture morphological analyses

External measurements of *indicus *and *tenuirostris *were taken to the nearest 0.1 mm using digital calipers. Measurements taken were: skull length, which is from distal tip of culmen to caudal end of cranium (only taken on specimens for which the rear cranium was not removed, determined by palpation); culmen length from the caudal edge of the cere; bill width and depth measured just proximal of the cere; greatest depth of the maxilla; nares length, which is the length of the nostril taken from the internal rim; gape width taken at the caudal limit of the rictus; bill length from gape, which is measured from the caudal limit of the rictus to the distal tip of the culmen; mandibular symphysis length measured from its caudal-most to distal-most limits; tail length from the base (junction of feather and skin) of the central rectrix; outer rectrix length from its base; length of ulna; wing and alula lengths from carpal joint; greatest tarsus length, proximal breadth, minimum shaft breadth, and distal breadth; lengths of each pedal digit measured in plantar aspect from the proximal limit of metatarsus I to the proximal limit of the claw; lengths of each claw measured from proximal to distal-most limits; and maximum width and depth of pes digit 3 claw.

We conducted Principal Components Analyses (PCA) for the morphological measurements to assess potential phenotypic distinctiveness of *indicus *and *tenuirostris *using a correlation matrix in Systat 8.0. Sexes were pooled as preliminary univariate statistical analyses and PCA showed no differences in size between the sexes, and because sex was not identified for many of the museum specimens for these two taxa. Measurements were also tested for significant differences using two-sample t-tests using the program Systat 8.0, with *P*-values ≤ 0.001 indicating significance after correcting for multiple comparisons [65].

## Authors' contributions

JAJ and DPM designed and developed the study. JAJ conducted most of molecular sequencing and phylogenetic analyses, and drafted the manuscript. HRLL provided samples of select taxa and contributed to molecular sequencing in the laboratory. PCR provided data on external measurements, statistical analyses, and text sections. All authors contributed to revising the final manuscript.

## Supplementary Material

Additional file 1 Sample information for *Gyps *and outgroup taxa used in this study. Click here for file
