# Supplementary material for: Systematics within Gyps vultures: a clade at risk
Source: BMC Evol Biol. 2006 Aug 23;6:65. doi: 10.1186/1471-2148-6-65 (PMC1569873; doi:10.1186/1471-2148-6-65)
Supplement: Additional file 1 — Sample information for Gyps and outgroup taxa used in this study. [file 1471-2148-6-65-S1.pdf]

Additional files

Additional file 1 – Sample information for *Gyps* and outgroup taxa used in this study.

					GenBank accession numbers		
DNA ID	Taxon	Sample/Museum ID[1]	Date collected	Location	cytB	ND2	CR
50	*Gyps bengalensis*	50	11-May-2002	Pakistan	DQ908977	AY987078	DQ908997
52	*Gyps bengalensis*	52	15-May-2002	Pakistan	DQ908975	AY987078	DQ908996
54	*Gyps bengalensis*	54	19-May-2002	Pakistan	DQ908977	AY987078	DQ908995
509	*Gyps bengalensis*	USNM 360567	00-Jan-1940	Cambodia	DQ908974	--	--
515	*Gyps bengalensis*	USNM 449794	10-Apr-1953	Thailand	DQ908977	--	--
516	*Gyps bengalensis*	USNM 278265	16-May-1918	Vietnam	DQ908976	--	--
518	*Gyps bengalensis*	FMNH 221723	11-Jan-1954	Nepal	DQ908975	--	--
201	*Gyps bengalensis*	GB3	2005	Cambodia	DQ908977	--	--
202	*Gyps bengalensis*	GB4	2005	Cambodia	DQ908979	--	--
203	*Gyps bengalensis*	GB5	2005	Cambodia	DQ908979	--	--
204	*Gyps bengalensis*	GB6	2005	Cambodia	DQ908978	--	--
205	*Gyps bengalensis*	GB7	9-May-2005	Cambodia	DQ908979	--	--
138	*Gyps bengalensis*[2]			Pakistan	AY987259	AY987078	DQ908998
--	*Gyps bengalensis*[3]			India	X86750	--	--
801	*Gyps indicus tenuirostris*	UMMZ 140548	31-Jan-1956	Assam, India	DQ908960	DQ908981	DQ908991
802	*Gyps indicus tenuirostris*	UMMZ 140549	31-Jan-1956	Assam,India	DQ908960	--	--
804	*Gyps indicus tenuirostris*	UMMZ 140551	14-Nov-1953	Assam,India	DQ908960	--	--
805	*Gyps indicus tenuirostris*	USNM 449793	4-Apr-1953	Thailand	DQ908960	--	--
806	*Gyps indicus tenuirostris*	FMNH 217888	2-Dec-1952	Nepal	DQ908960	DQ908982	DQ908991
807	*Gyps indicus tenuirostris*	FMNH 228896	5-Feb-1947	Uttar Pradesh, India	DQ908960	--	--
808	*Gyps indicus tenuirostris*	FMNH 228897	15-Feb-1947	Uttar Pradesh, India	DQ908961	--	--
809	*Gyps indicus tenuirostris*	FMNH 228899	7-Feb-1947	Uttar Pradesh, India	DQ908960	--	--

301	*Gyps indicus tenuirostris*	GT1	2005	Cambodia	DQ908960	--	--
302	*Gyps indicus tenuirostris*	GT2	2005	Cambodia	DQ908960	--	--
303	*Gyps indicus tenuirostris*	GT3	4-May-2005	Cambodia	DQ908960	--	--
304	*Gyps indicus tenuirostris*	GT4	8-May-2005	Cambodia	DQ908960	--	--
305	*Gyps indicus tenuirostris*	GT5	9-May-2005	Cambodia	DQ908960	--	--
833	*Gyps indicus indicus*	FMHN 228895	18-Feb-1938	Bombay, India	DQ908971	DQ908989	DQ909004
835	*Gyps indicus indicus*	FMNH 228901	12-Dec-1946	Madhya Pradesh, India	DQ908971	--	--
836	*Gyps indicus indicus*	FMNH 228904	23-Dec-1946	Madhya Pradesh, India	DQ908971	DQ908989	DQ909005
810	*Gyps himalayensis*	UMMZ 140544	1-Feb-1956	Assam, India	DQ908962	DQ908983	DQ908992
811	*Gyps himalayensis*	UMMZ 140545	1-Feb-1956	Assam, India	DQ908962	--	--
812	*Gyps himalayensis*	UMMZ 140547	5-Apr-1956	Assam, India	DQ908963	--	--
813	*Gyps himalayensis*	FMNH 218931	1-Jan-1954	Nepal	DQ908962	DQ908983	DQ908992
814	*Gyps himalayensis*	USNM 304560	2-Jun-1923	China	DQ908962	--	--
815	*Gyps himalayensis*	MCZ 142716	21-Jul-1929	Sichuan, China	DQ908964	--	--
108	*Gyps fulvus fulvus*	Case 2	20-Mar-2004	Pakistan	AY987261	--	--
109	*Gyps fulvus fulvus*	Case 3	20-Mar-2004	Pakistan	AY987261	DQ908985	DQ908993
817	*Gyps fulvus fulvus*	UMMZ 140546	11-Dec-1953	Assam, India	DQ908970	--	--
818	*Gyps fulvus fulvus*	FMNH 228890	3-Oct-1942	Luristan, Iran	DQ908968	--	--
821	*Gyps fulvus fulvus*	MCZ 70787	?	Palestine	AY987261	--	--
822	*Gyps fulvus fulvus*	MCZ 92693	13-Apr-1911	Kazakhstan	AY987261	--	--
828	*Gyps fulvus fulvus*	AMNH 535635	25-Jan-1892	South Spain	AY987261	--	--
829	*Gyps fulvus fulvus*	UMMZ 25013	?	Asia Minor	DQ908967	--	--
831	*Gyps fulvus fulvus*	FMNH 228892	16-Jan-1942	Bakhtiari, Iran	AY987261	--	--
89	*Gyps fulvus fulvus*	A1235	26-Jan-2003	Gambia	AY987261	DQ908986	DQ908994
90	*Gyps fulvus fulvus*	A1236	26-Jan-2003	Gambia	AY987261	--	--
91	*Gyps fulvus fulvus*	A1237	28-Jan-2003	Gambia	DQ908969	--	--
B19181	*Gyps fulvus fulvus*[2]			Gambia	AY987261	AY987080	DQ908994
816	*Gyps fulvus fulvescens*	UMMZ 78123	29-Jan-1937	Haryana, India	DQ908966	--	--

819	*Gyps fulvus fulvescens*	FMNH 268077	25-Nov-1961	Nepal	DQ908965	DQ908984	DQ908992
324	*Gyps coprotheres*	DWC-9	4-Jun-2003	South Africa	AY987262	--	--
325	*Gyps coprotheres*	DWC-11	4-Jun-2003	South Africa	AY987262	AY987081	DQ908999
DWC-10	*Gyps coprotheres*[2]			South Africa	AY987262	AY987081	DQ908999
--	*Gyps coprotheres*[3]			South Africa	X86751	--	--
326	*Gyps africanus*	DWC-12	4-Jun-2003	(not given)	DQ908972	DQ908988	DQ909003
21	*Gyps africanus*[2]			(not given)	AY987263	AY987082	DQ909002
--	*Gyps africanus*[3]			South Africa	X86748	--	--
92	*Gyps rueppellii*	A1231	28-Jan-2003	Gambia	DQ908973	DQ908987	DQ909001
A1119	*Gyps rueppellii*[2]			Gambia	AY987260	AY987079	DQ909000
83	*Necrosyrtes monachus*	A1234	14-Jan-2003	Gambia	DQ908980	DQ908990	--
A1234	*Necrosyrtes monachus*[2]			Gambia	AY987259	AY987077	DQ909006
DWC-20	*Sarcogyps calvus*[2]			South Africa	AY987264	AY987083	DQ909007
1903	*Aegypius monachus*[2]			(not given)	AY987266	AY987085	--
--	*Aegypius monachus*[3]			(not given)	X86739	--	--
T-2046	*Torgos tracheliotos*[2]			South Africa	AY987267	AY987086	--
--/130316	*Trigonoceps occipitalis*[2,3]			Africa	X86762	AY987084	--

[1]AMNH, American Museum of Natural History, NY; DWC, De Wildt Cheetah & Wildlife Reserve, Pretoria, South Africa; FMNH, Field Museum of Natural History, Chicago; MCZ, Museum of Comparative Zoology, Harvard Univ.; UMMZ, Univ. of Michigan Museum of Zoology; USNM, United States National Museum, Washington D.C.

[2] Lerner HRL & DP Mindell 2005 *Mol. Phylogenet. Evol.* 37:327-346.

[3] Seibold I & AJ Helbig 1995 *Phil. Trans. R. Soc. Lond.* B 350:163-178.
